# Novel Ultralight-Weight Complex Concentrated Alloys with High Strength

**DOI:** 10.3390/ma12071136

**Published:** 2019-04-08

**Authors:** Yuefei Jia, Yandong Jia, Shiwei Wu, Xindi Ma, Gang Wang

**Affiliations:** Laboratory for Microstructures, Institute of Materials, Shanghai University, Shanghai 200444, China; JiaYF710@163.com (Y.J.); wushiwei@shu.edu.cn (S.W.); melodyxd@126.com (X.M.)

**Keywords:** ultralight-weight complex concentrated alloys, high specific strength, low Young’s modulus

## Abstract

To explore a novel high strength and low modulus ultralight-weight complex concentrated alloys (ULW-CCAs), a series of light alloys are designed and explored based on some low-density and low modulus elements, such as Al, Li, Mg, Ca, Si, and Y. An Al_19.9_Li_30_Mg_35_Si_10_Ca_5_Y_0.1_ (at %) CCA with a high specific strength of 327 KPa·m^−3^ is successfully developed. After adjusting the composition, the Al_15_Li_35_Mg_48_Ca_1_Si_1_ CCA with the good compressive plasticity is successfully developed. The Al_15_Li_38_Mg_45_Ca_0.5_Si_1.5_ and Al_15_Li_39_Mg_45_Ca_0.5_Si_0.5_ CCAs exhibit good plasticity of >45%, and >60%, respectively. These ULW-CCAs show the high specific strength, good ductility, and low Young’s modulus, as compared with the previously reported CCAs.

## 1. Introduction

Lightweight is of importance for developing advanced alloys used in aircraft, military, and electronic industry, etc. [1]. The light-weight alloys, based on Al, Mg, and Ti, usually have low density and high strength, which are attractive in the structural materials field [2]. However, traditional light-weight alloys are usually based on one dominant element, which limits the exploration of new light-weight alloys. High-entropy alloys (HEAs) [3] or complex concentrated alloys (CCAs) [4,5] are a new emerging class of potentially structural materials [6], which contain multi-principle elements [5]. The innovative concepts of multi-principle elements change the paradigm of traditional-alloy design strategy, and open up new opportunities for the discovery of new alloys. In other words, some unknown compositional region of multicomponent phase diagrams could be explored based on this concept [7]. The developed CCAs have attracted increased attention because of their unique compositions, microstructures, and outstanding properties, such as ultrahigh fracture toughness [8], high strength [9,10,11], good resistances for fatigue, corrosion, and oxidation [12,13,14,15].

To date, a few studies have reported on the development of low-density CCAs with a high strength based on many light elements, such as Al, Be, Li, Mg, Sc, Si, Sn, Cu, Ti, and Zn [16,17,18,19,20,21]. For example, a light-weight Al_20_Li_20_Mg_10_Sc_20_Ti_30_ CCA (with a density of 2.67 g·cm^−3^) was fabricated by mechanical alloying, which displayed a high Vickers hardness (HV) of ~5.5 GPa [20,21]. An Al_20_Be_20_Fe_10_Si_15_Ti_35_ CCA shows a density and a hardness (HV) of 3.91 g·cm^−3^ and ~8.9 GPa, respectively [21]. A series of Mg_x_(MnAlZnCu)_100−x_ CCAs were developed, which exhibit high compressive strengths of 400–500 MPa and low densities ranged from 4.29 to 2.20 g·cm^−3^, respectively [18]. These Mg_x_(MnAlZnCu)_100−x_ CCAs present a multi-phase structure, especially, a hexagonal close-packed (HCP) phase and an Al-Mn icosahedral quasicrystal phase are predominated [17]. Furthermore, some beryllium-bearing low-density CCAs, such as BeCoMgTi and BeCoMgTiZn, which were synthesized by mechanical alloying, exhibit a complete amorphous phase [19]. However, these light-weight CCAs usually have some major drawbacks. For instances, their density is larger than 2 g·cm^−3^, which cannot be further decreased; they exhibit a brittle nature and hard processability due to big differences between melting and boiling temperatures from different constitute elements. These disadvantages limit their applications [7]. Therefore, the development of ultralight-weight complex concentrated alloys (ULW-CCAs) to approach a high specific strength is important for the structural material field.

In the present study, ULW-CCAs with the density being lower than 1.8 g∙cm^−3^, high strength, low modulus, and relative high ductility are developed. The new developed ULW-CCAs in this study possess higher specific strength and ductility as compared with the previously reported LW-CCAs. Our novel ULW-CCAs would provide more potential applications in the field of light-weight structure materials.

## 2. Experimental Methods

### 2.1. Compositional Elements for Novel ULW-CCAs

**Figure 1:**
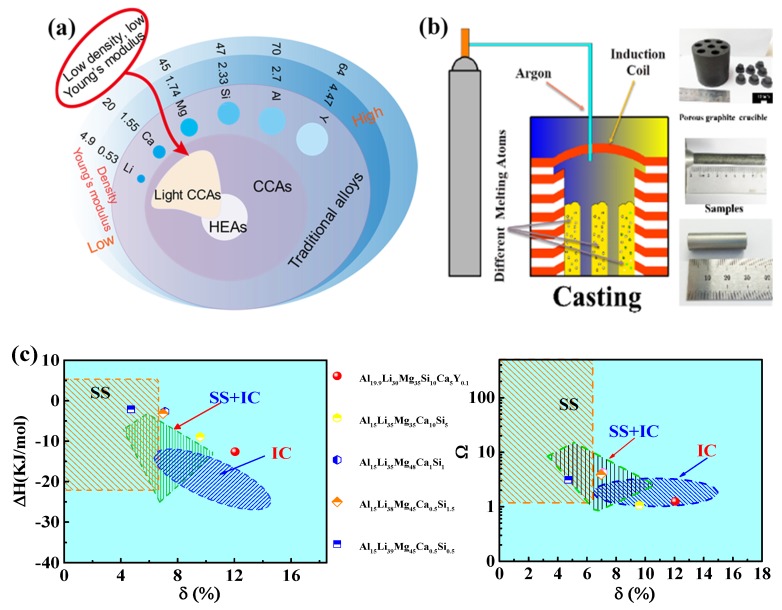
Composition design and synthesis of the ultralight-weight complex concentrated alloys (ULW-CCAs). (**a**) Schematic of material design. (**b**) Casting process. (**c**) Phase constituent prediction maps (the relationship between parameters of *δ*, ∆*H_mix_*, and Ω.

Generally, the lightest metal elements of Li and Mg are chosen to design novel ULW-CCAs. Other elements containing the special properties, such as Ca, Si, and Y are also considered to be adopted in ULW-CCAs. Thus, six elements from low density to high density (Li, Ca, Mg, Si, Al, and Y, as shown in Figure 1a) are used to design ULW-CCAs. The physical and chemical parameters of the six elements are summarized in Table 1.

### 2.2. Theoretical Design

Phase formation is important for designing CCAs. The concepts of CCAs suggest that disordered solid solutions are more stable than the ordered intermetallic compounds [6]. Although previous studies suggested that, due to the effect of entropy, a compositional complexity does not bring out a microstructural complexity [10,22], there are no universal theories for the phase-formations in CCAs with the compositional complexity. The calculation of phase diagram is one of the methods [23,24,25,26] to predict the phase stability. Comprehensively considering the differences of the atomic sizes, *δ*, the enthalpy of mixing, Δ*H_mix_*, and the ideal entropy of mixing of the alloys also can predict the criteria for the phase stability based on the Hume-Rothery rule [16,22,27,28,29,30,31,32]. The parameter of Ω is defined as the entropy of mixing, times, and the average melting temperature of the elements divided by the enthalpy of mixing [27]. *δ* is the mean square deviation of the atomic size of elements [30]. Moreover, the entropy of mixing, ∆*S_mix_*, ∆*H_mix_*, the Pauling electronegativity difference, ∆*χ*, and the valence-electron concentration, *VEC*, are also used to characterize the phases formation [27,28,33], which indicate that *δ* vs. ∆*H_mix_*, *δ* vs. Ω, *δ* vs. ∆*χ*, *δ* vs. *VEC* could be used to predict the formation of solid-solution phases for CCAs.

The following equations are adopted to define the parameters for CCAs [16,20,27,28,29,31,32,33]:

(1) $$∆S_{mix}=-R\sum_{i}^{n}(c_{i}\ln c_{i}),$$

(2) $$∆H_{mix}=\sum_{i=1,i\neq j}^{n}\Omega_{ij}c_{i}c_{j},$$

where *c_i_* and *c_j_* are the atomic percentages of the *i*-th and *j*-th components, respectively. Ω*_ij_* (= $4∆H_{AB}^{mix}$) is a regular solution-interaction parameter between the *i*-th and *j*-th elements. $∆H_{AB}^{mix}$ is an enthalpy of mixing of binary alloys, and *R* is the gas constant (8.314 J·mol^−1^·K^−1^).

(3) $$\Omega =\frac{T_{m}∆S_{mix}}{\left|∆H_{mix}\right|},$$

(4) δ=∑i=1nci(1−rir¯)2,

where $\bar{r}$ is the average atomic radius, and *r_i_* is the atomic radius, *T_m_* ($=\sum_{i=1}n_{c_{i}}{\left(T_{m}\right)}_{i})$ is the melting temperature of an *n*-elements alloy, and ${\left(T_{m}\right)}_{i}$ is the melting point of the *i*-th component of an alloy.

(5) $$∆\chi =\sqrt{\sum_{i=1}^{n}c_{i}{\left(\chi_{i}-\bar{\chi }\right)}^{2}},$$

(6) $$VEC=\sum_{i}^{n}c_{i}{\left(VEC\right)}_{i},$$

where $\bar{\chi }=\sum_{i=1}^{n}c_{i}\chi_{i}$ is the Pauling electronegativity for the *i*-th component, and (*VEC*)*_i_* is the *VEC* of the *i*-th element. Basically, solid solutions can form when the *δ* value is small, i.e., *δ* < 6.6%, and the Δ*H_mix_* value is either slightly positive or insignificantly negative, i.e., −11.6 < Δ*H_mix_* < 3.2 kJ/mol, or Ω ≥1.1 [27]. On the contrary, the amorphous phase can form when the *δ* value is larger than 6.4%, and the Δ*H_mix_* value is noticeably negative, i.e., Δ*H_mix_* < −12.2 kJ/mol [22,32]. The corresponding phase-constituent prediction maps are shown in Figure 1c, in which ‘‘SS’’ indicates a region where only a solid solution can form for the multicomponent alloys, ‘‘IC’’ represents that the multicomponent alloys mainly contain intermetallic compounds and other ordered phases, ‘‘S + I’’ implies that both solid solutions and ordered compounds can form. In the present study, according to the Hume-Rothery rule [20,21,22,24,26,27,28], several novel ULW-CCAs are designed in a range of the ∆*S_mix_* value, i.e., from 1.075*R* to 1.514*R*, which include Al_19.9_Li_30_Mg_35_Si_10_Ca_5_Y_0.1_, Al_15_Li_35_Mg_35_Ca_10_Si_5_, Al_15_Li_35_Mg_48_Ca_1_Si_1_, Al_15_Li_38_Mg_45_Ca_0.5_Si_1.5_, and Al_15_Li_39_Mg_45_Ca_0.5_Si_0.5_ (at.%) ULW-CCAs [3]. The phase compositions of these five ULW-CCAs are summarized in Figure 1c, in which the Al_19.9_Li_30_Mg_35_Si_10_Ca_5_Y_0.1_ and Al_15_Li_35_Mg_35_Ca_10_Si_5_ ULW-CCAs may be the intermetallic compounds, the Al_15_Li_35_Mg_48_Ca_1_Si_1_ and Al_15_Li_38_Mg_45_Ca_0.5_Si_1.5_ ULW-CCAs may contain the solid-solution phases and the intermetallic compounds, and the Al_15_Li_39_Mg_45_Ca_0.5_Si_0.5_ ULW-CCA may be the solid-solution phase. All the phase-formation parameters are listed in Table 2.

### 2.3. Experiment Process

Al-Si master alloys with 50 wt.% Si, and commercially pure Al, Mg, Li, Ca, and Y (purity > 99.9%) were selected as raw materials. Five samples with the compositions of Al_19.9_Li_30_Mg_35_Si_10_Ca_5_Y_0.1_, Al_15_Li_35_Mg_35_Ca_10_Si_5_, Al_15_Li_35_Mg_48_Ca_1_Si_1_, Al_15_Li_38_Mg_45_Ca_0.5_Si_1.5_, and Al_15_Li_39_Mg_45_Ca_0.5_Si_0.5_ were prepared by induction melting in an argon atmosphere and a graphite crucible. The melted alloys were then cast into a copper mold to form cylindrical rods with a diameter of 10 mm (Figure 1b).

The cylindrical samples were cut into pieces with a diamond saw and then were ground, polished, and etched (in a 2.5% nitric acid-methanol). The structures were identified in a Gigaku D\max-2550 X-ray diffractometer (XRD, Rigaku Company, Tokyo, Japan) with a Cu-Kα radiation. The microstructures of the ULW-CCAs were investigated by an Apollo 300 scanning electron microscopy (SEM, CamScan Company, Waterbeach, UK) equipped with the backscattering electron (BSE) detector, and a JEOL 2100 type transmission electron microscope (TEM, JEOL Company, Tokyo, Japan). The compositions of the constituents were analyzed by energy dispersion spectrum (EDS) in the Apollo 300 SEM. Note that Li could not be detected by EDS. TEM samples were prepared by a focus ion beam (600i, FEI Company, Diepoldsau, Switzerland). Compression tests at a strain rate of 1 × 10^−4^ s^−1^ were performed in an MTS CMT 5205 machine. The compression samples were shaped into a geometric size of Φ6 mm × 12 mm. For the nanoindentation experiment, five ULW-CCAs wafers, i.e., Al_19.9_Li_30_Mg_35_Si_10_Ca_5_Y_0.1_, Al_15_Li_35_Mg_35_Ca_10_Si_5_, Al_15_Li_35_Mg_48_Ca_1_Si_1_, Al_15_Li_38_Mg_45_Ca_0.5_Si_1.5_, and Al_15_Li_39_Mg_45_Ca_0.5_Si_0.5_, with a diameter of 10 mm and a thickness of 4 mm were fabricated. All the samples were polished and etched to clearly show distinct phases. The polished and etched samples for nanoindentation tests were loaded at a maximum load of 8 mN (Tribo indenter, Hysitron Company, Minneapolis, MN, USA). The density was measured by Archimedes’ principle in absolute alcohol (purity of 99.9%).

## 3. Results

**Figure 2:**
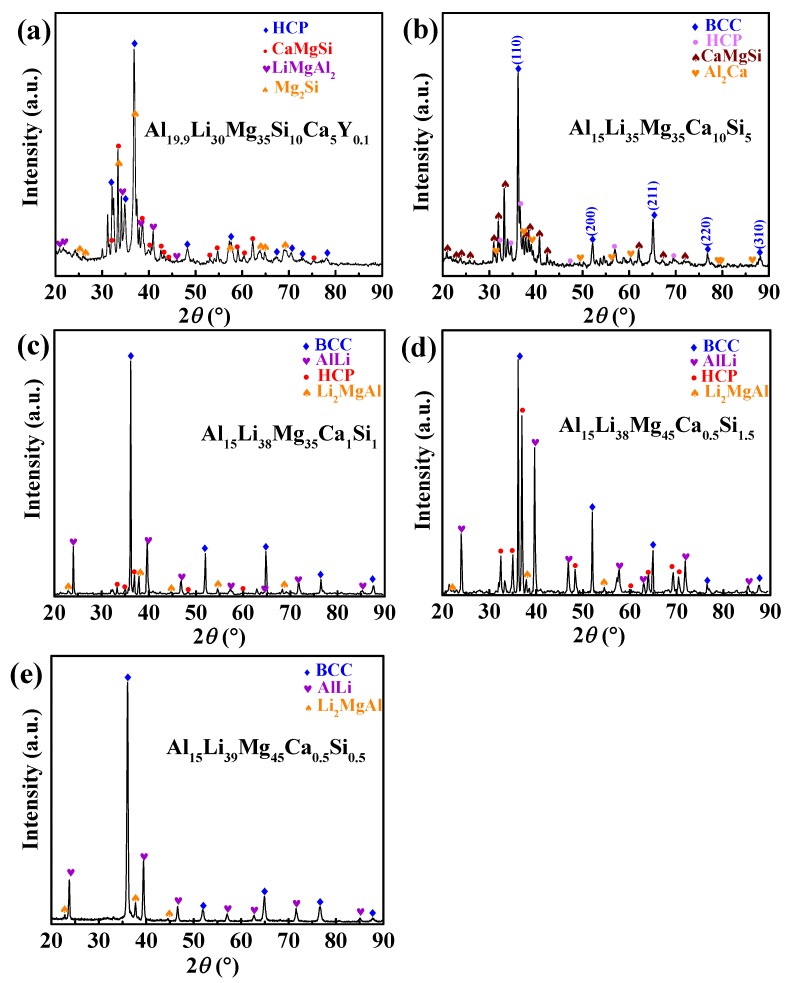
X-ray diffraction patterns of five ULW-CCAs. (**a**) Al_19.9_Li_30_Mg_35_Si_10_Ca_5_Y_0.1_ ULW-CCA; (**b**) Al_15_Li_35_Mg_35_Ca_10_Si_5_ ULW-CCA; (**c**) Al_15_Li_35_Mg_48_Ca_1_Si_1_ ULW-CCA; (**d**) Al_15_Li_38_Mg_45_Ca_0.5_Si_1.5_ ULW-CCA; (**e**) Al_15_Li_39_Mg_45_Ca_0.5_Si_0.5_ ULW-CCA.

The XRD patterns of five types of ULW-CCAs (Al_19.9_Li_30_Mg_35_Si_10_Ca_5_Y_0.1_, Al_15_Li_35_Mg_35_Ca_10_Si_5_, Al_15_Li_38_Mg_45_Ca_1_Si_1_, Al_15_Li_35_Mg_48_Ca_0.5_Si_1.5_, andAl_15_Li_39_Mg_45_Ca_0.5_Si_0.5_) are shown in Figure 2. The XRD pattern indicates that the near equiatomic Al_19.9_Li_30_Mg_35_Si_10_Ca_5_Y_0.1_ ULW-CCA contains HCP solid solution and intermetallic (IM) phases (Figure 2a). Furthermore, more than four phases coexist in this ULW-CCA. The Al_15_Li_35_Mg_35_Ca_10_Si_5_ ULW-CCA consists of four phases, i.e., a body-centered cubic (BCC) solid solution, an HCP solid solution, Al_2_Ca and CaMgSi intermetallics (Figure 2b). The phase structures of the Al_15_Li_38_Mg_45_Ca_1_Si_1_, Al_15_Li_35_Mg_48_Ca_0.5_Si_1.5_, and Al_15_Li_39_Mg_45_Ca_0.5_Si_0.5_ ULW-CCAs are almost the same (Figure 2c–e). The dominant phase of these three alloys is a BCC solid solution. Additionally, the AlLi and Li_2_MgAl phases can also be easily identified in these three ULW-CCAs.

The densities of the ULW-CCAs are measured, which are close to the theoretical values, *ρ_theo_*, in Table 2. The theoretical density is estimated by a rule of mixture assumption of a disordered solid solution, as given by $\rho_{theo}=\frac{\sum_{i}^{n}c_{i}M_{i}}{\sum_{i}^{n}c_{i}V_{i}}$, where *c_i_*, *M_i_* and *V_i_* are the atomic fractions, molar mass, and molar volume of each constituent element, and *n* is the total number of elements [23]. As shown in Table 2, the densities of these ULW-CCAs mainly ranged from 1.46 to 1.70 g·cm^−3^, which are significantly smaller than those of previously reported CCAs [16,17,18,20,21,34,35,36]. The lowest density of the ULW-CCA already approaches 1.46 ± 0.05 g·cm^−3^.

**Figure 3:**
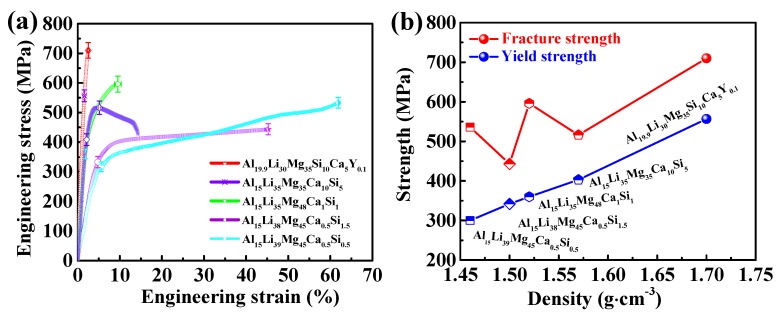
Mechanical properties of five ULW-CCAs. (**a**) Engineering stress–strain curves. (**b**) Relationships between yield strength, fracture strength, and density.

The compression stress–strain curves of the ULW-CCAs are plotted in Figure 3a. The relationships between the strengths, including yield strength and fracture strength and the densities of the ULW-CCAs are shown in Figure 3b. For the alloy system of AlLiMgCaSi, their strengths almost linearly increase with increasing the densities by adjusting the atomic percentages of the constitutive elements (Figure 3b). When Y is doped in the AlLiMgCaSi, i.e., the Al_19.9_Li_30_Mg_35_Si_10_Ca_5_Y_0.1_ ULW-CCA, it exhibits the highest fracture strength of 710 ± 26 MPa and yield strength of 556 ± 20 MPa without significant plastic strain in Figure 3a. Al_15_Li_35_Mg_35_Ca_10_Si_5_ ULW-CCA also shows high fracture strength of 516 ± 33 MPa and high yield strength 418 ± 29 MPa. Changing the atomic percentage of the elements in the AlLiMgCaSi system causes that the Al_15_Li_38_Mg_45_Ca_0.5_Si_1.5_ ULW-CCA approaches a good combination of mechanical property, i.e., a relatively high yield strength of 342 ± 19 MPa, a low density of 1.50 ± 0.05 g cm^−3^ and a high compressive ductility of >45%. The Al_15_Li_39_Mg_45_Ca_0.5_Si_0.5_ ULW-CCA also show a yield strength of 300 ± 33 MPa, a very low density of 1.46 ± 0.05 g cm^−3^ and a good compressive ductility of >60%. Nevertheless, the Al_15_Li_35_Mg_48_Ca_1_Si_1_ ULW-CCA demonstrates not only a high fracture strength of 596 ± 27 MPa and a yield strength of 360 ± 16 MPa, but also exhibits a good ductility of 9.5 ± 0.8%, as compared with the values in the Al_15_Li_38_Mg_45_Ca_0.5_Si_1.5_ and Al_19.9_Li_30_Mg_35_Si_10_Ca_5_Y_0.1_ ULW-CCAs.

**Figure 4:**
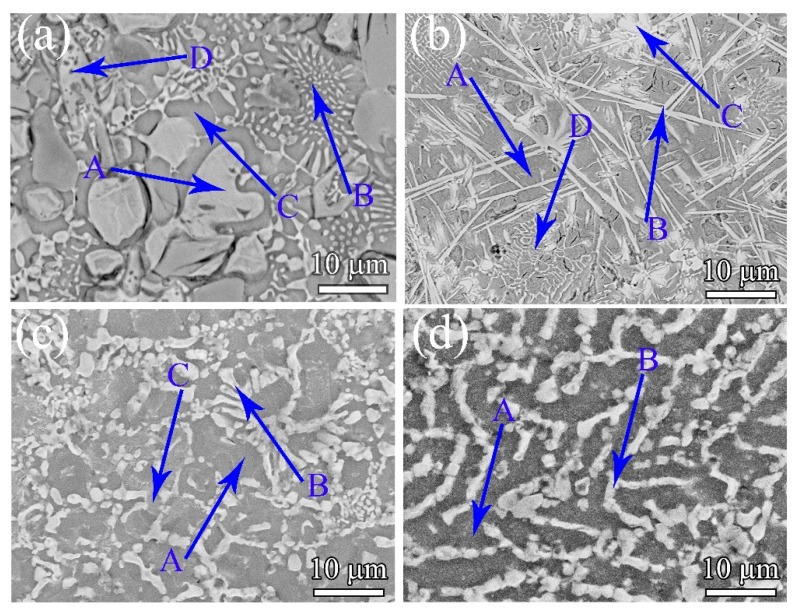
Microstructures of (**a**) Al_19.9_Li_30_Mg_35_Ca_10_Si_5_Y_0.1_ ULW-CCA, (**b**) Al_15_Li_35_Mg_35_Si_5_Ca_10_ ULW-CCA, (**c**) Al_15_Li_38_Mg_35_Ca_0.5_Si_1.5_ ULW-CCA and (**d**) Al_15_Li_39_Mg_35_Ca_0.5_Si_0.5_ ULW-CCA.

The BSE image of the Al_19.9_Li_30_Mg_35_Ca_10_Si_5_Y_0.1_ ULW-CCA is shown in Figure 4a, indicating that the microstructure is composed of four different regions, as marked by A, B, C, and D. According to the EDS analyses listed in Table 3, the Al-enriched region corresponds to region A. The phase in this region is identified as the LiMgAl_2_ phase and its volume fraction is approximately 31%. Region B and region C are identified as the Mg_2_Si phase and the unknown phase, respectively. Furthermore, D is deduced to be the CaMgSi phase comprised of 30.5 at.% Mg, 39.4 at.% Si, and 30.1 at.% Ca.

The BSE image of the Al_15_Li_35_Mg_35_Si_5_Ca_10_ ULW-CCA, as shown in Figure 4b, indicates that the dark-grey matrix (region A in Figure 4b) represents a β-Mg (BCC) phase. The volume fraction of the β-Mg phase is estimated to be 45–57%. According to the EDS results in Table 3 and the XRD pattern (Figure 2b), the phase structure of region B is characterized to be the Al_2_Ca phase. Likewise, the structure with bulk shape (region C in Figure 4b), and the reticulate-like phase (region D in Figure 4b) are identified to be the HCP phase and the CaMgSi phase, respectively.

The morphology of the Al_15_Li_35_Mg_48_Ca_0.5_Si_1.5_ ULW-CCA is shown in Figure 4c. The Al_15_Li_35_Mg_48_Ca_0.5_Si_1.5_ ULW-CCA is made up of the BCC solid solution (β-Mg), the HCP solid solution (α-Mg) and the AlLi phase. The dark region (region A in Figure 4c) corresponds to the β-Mg BCC solid solution phase. The lath-like morphology (region B in Figure 4c) is the AlLi phase with an average size of 1–15 μm, and the volume fraction of 28–32%. Region C in Figure 4c could be the HCP solid solution according to the EDS results (Table 3). As shown in Figure 4d, the microstructure of the Al_15_Li_39_Mg_48_Ca_0.5_Si_0.5_ ULW-CCA consists of two primary phases. The matrix, i.e., region A in Figure 4d, consists of the BCC solid solution and the Li_2_MgAl phase. In addition, based on the XRD (Figure 2e) and EDS results (Table 3), the lath-like phase (region B in Figure 4d) is identified as the AlLi phase. The AlLi phase with a volume fraction of 25–28% is uniformly distributed in the matrix. Both the Al_15_Li_35_Mg_48_Ca_0.5_Si_1.5_ and Al_15_Li_39_Mg_48_Ca_0.5_Si_0.5_ ULW-CCAs display a dendritic structure, which is divided by the net-like interdendritic structure.

**Figure 5:**
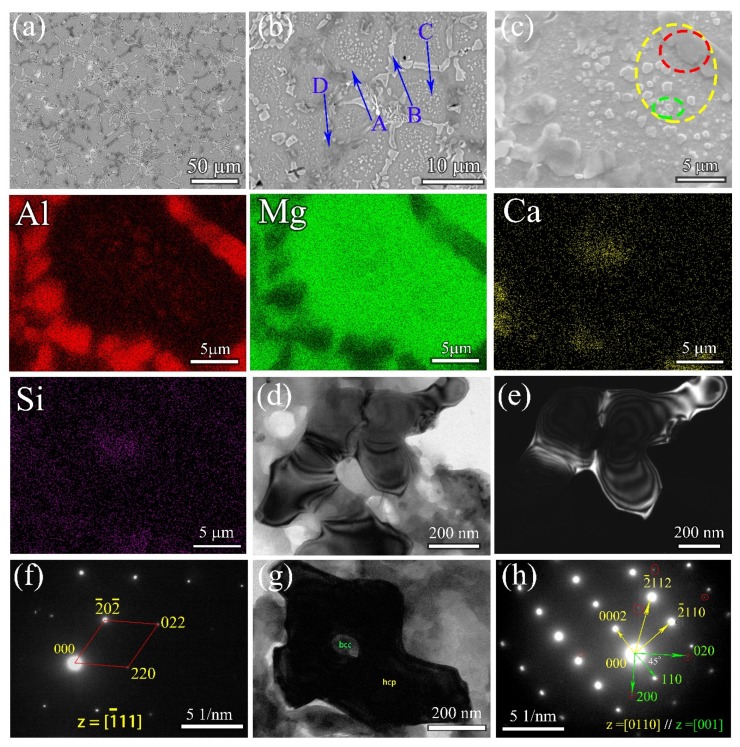
Microstructures of Al_15_Li_35_Mg_48_Ca_1_Si_1_ ULW-CCA. (**a**–**c**) are BSE images (from low to high magnification); “Al”, “Mg”, “Ca” and “Si” are the EDS mapping of (**c**); (**d**) The bright-field image (BF) of red circled phase in (**c**); (**e**) The dark-field (DF) image of the red circled phase in (**c**); (**f**) The selected area diffraction pattern of (**e**). (**g**) The bright-field (BF) for submicron particle marked by green circle region in (**c**). (**h**)The selected area diffraction patterns of dark area in (**g**).

According to Figure 3, the Al_15_Li_38_Mg_45_Ca_1_Si_1_ ULW-CCA has a good comprehensive property, i.e., the high strength accompanied by the good ductility. Thus, the microstructure of the Al_15_Li_38_Mg_45_Ca_1_Si_1_ ULW-CCA is further characterized. The BSE images of the Al_15_Li_38_Mg_45_Ca_1_Si_1_ ULW-CCA are shown in Figure 5a–c. The EDS maps are also given in Figure 5. The microstructure consists of the matrix (region A in Figure 5b), the lath-like phase (region B and region C in Figure 5b), and submicron-size particles (region D in Figure 5b). Based on the EDS map, the lath-like phase (region B) is an Al-enriched phase (Table 3). Combined with the X-ray result of this CCA (Figure 2c), the TEM observation indicates that the lath-like phase could be referred as the AlLi phase (Figure 5d–f), and region D is the Li_2_MgAl phase (Figure 5b). The size of the AlLi phase is 5–20 μm. The matrix is the BCC phase that is determined by the selected-area electron diffraction (SAED) pattern (Figure 5h) In addition, some submicron-sized particles with the HCP structure (Figure 5b,c) are embedded in the BCC structure matrix and are surrounded by the lath-like Al-Li and Li_2_MgAl phases (Figure 5c).

**Figure 6:**
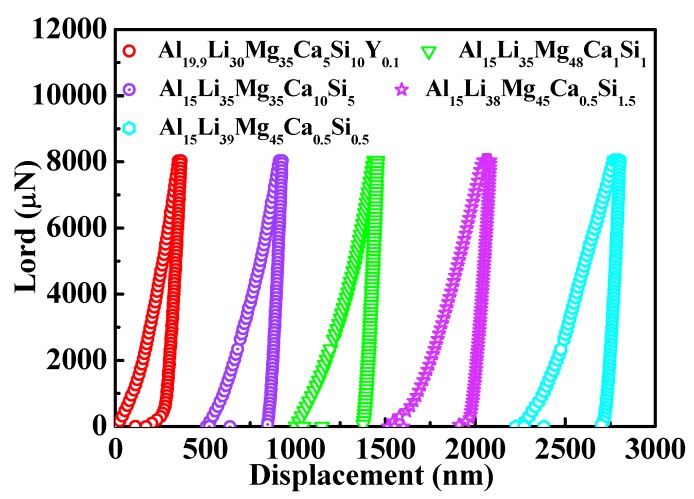
Representative load-displacement curve of nanoindentation tests for five ULW-CCAs.

Nanoindentation tests were carried out to characterize the hardness and Young’s modulus of different phases in the ULW-CCAs. Considering that the measured area of the nanoindentation test is localized in a very small region with a diameter of several hundred nanometers, the hardness, and elastic modulus values must be very scattering. Furthermore, because the nanoindentator tip touching the area usually can slightly deviate from the target area during the experiment, the nanoindentation tests for each ULW-CCA were repeatedly carried out to exclude the odd values. In our study, the nanoindentation tests were repeated ten times to exclude the occasional result. The load-displacement curves of the nanoindentation tests for each ULW-CCA are representatively shown in Figure 6. Based on the load-displacement curve, the hardness and Young’s modulus values are evaluated [37].

## 4. Discussion

**Figure 7:**
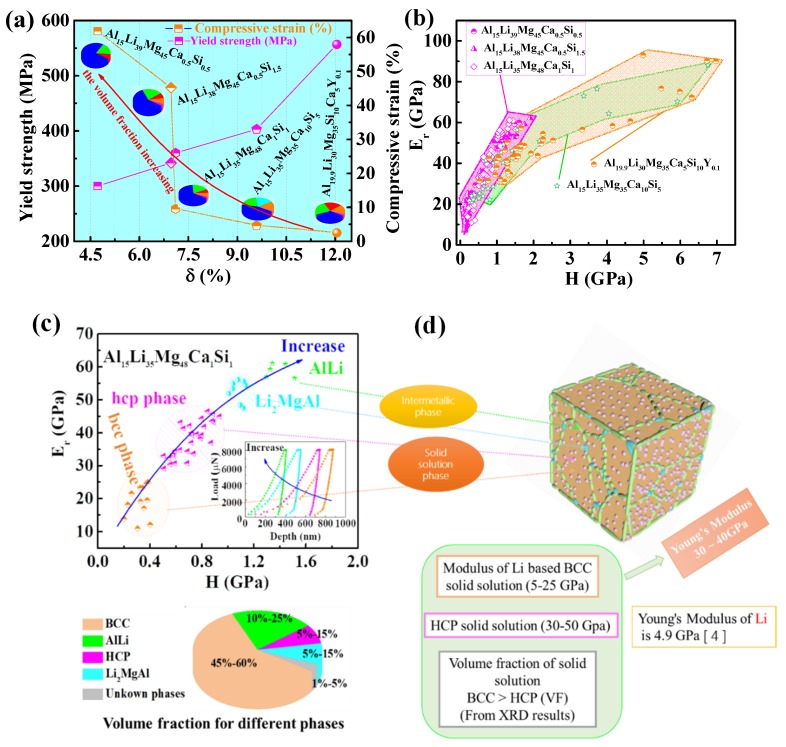
(**a**) Relationship between yield strength, compressive strain, volume fractions of solid-solution phases and *δ* (%). (**b**) Young’s modulus as a function of hardness for five ULW-CCAs. (**c**) The relationship between Young’s modulus and hardness for different solid-solution phases and intermetallic compounds. (**d**) Structure model of the Al_15_Li_35_Mg_48_Ca_1_Si_1_ ULW-CCA.

The compressive strain and the yield strength as functions of the *δ* value of five ULW-CCAs are summarized in Figure 7a that also includes the volume fraction of different phases, i.e., the solid solution and the intermetallic compounds. The volume fraction of solid solution, marked by the blue part in the pie chart (Figure 7a), increases from the Al_19.9_Li_30_Mg_35_Ca_10_Si_5_Y_0.1_ to Al_15_Li_39_Mg_45_Ca_0.5_Si_0.5_ ULW-CCAs, which is calculated from the SEM results. Meanwhile, with increasing the volume fraction of solid solution, the *δ* value decreases. It can be seen that our ULW-CCAs are closed to the “SS” region (the *δ* value is small) in Figure 1c with the simpler phase constitution and the larger volume fraction of solid solution, as compared with those alloys far from the SS region (the *δ* value is large). The compressive ductility is improved by increasing the volume fraction of solid solution, as given in Figure 3a and Figure 7a, while the yield strength of these ULW-CCAs decreases with an increase in the volume fraction of solid solution. Therefore, the solid solution phases play a significant role in the plastic deformation in the ULW-CCAs.

The relationship between Young’s modulus and the hardness is shown in Figure 7b. Both the BCC solid solution and the HCP solid solution exhibit low Young’s modulus and low hardness, as shown in Figure 7c. In contrast, the values of modulus and hardness for different intermetallic compounds are all higher than those of the solid solutions. As shown in Figure 7d, Young’s modulus of pure Li is approximately 4.9 GPa. The BCC solid solution is a Li-rich phase with a low modulus of 10–25 GPa according to the nanoindentation results, which corresponds to the orange region in Figure 7d, and the 3D diagram of the Al_15_Li_35_Mg_48_Ca_1_Si_1_ ULW-CCA in Figure 7c,d. Moreover, the HCP solid solution is represented by the pink balls embedded into the BCC matrix in Figure 7d. The HCP solid solution also has a low modulus of 30–50 GPa according to Figure 7c. A number of intermetallic compounds surround the BCC matrix, which is represented by the green areas (the AlLi phase) and the cerulean areas (the Li_2_MgAl phase). These intermetallic compounds show higher moduli than that of the solid solution. However, the volume fraction of solid solutions including the BCC and HCP phases is much larger than that of intermetallic compounds. The volume fraction of the BCC solid solution is also larger than that of the HCP solid solution. Thus, the low Young’s modulus of the Al_15_Li_35_Mg_48_Ca_1_Si_1_ ULW-CCA is mainly dominated by the large volume fraction of solid solution, especially the volume fraction of the BCC solid solution. Accordingly, the high volumes fraction of solid solutions including the BCC and HCP phases can lead to a low modulus. The low densities (1.46~1.70 g·cm^−3^), the high fracture strength (>450 MPa), and low Young’s modulus of the ULW-CCAs are associated with the mixture of primary characteristics of all the constituent elements and the constituent phases in each ULW-CCA [29]. For example, the low density of each ULW-CCA comes from the mixture of its constituent light elements, and the high fracture strength is from the overall contribution of the constituent phases, which include the effect of phase shape, phase distribution, phase boundaries, and properties of each phase. For our designed ULW-CCAs, the phase composition is complex. The morphology of the intermetallic compounds is irregular. Thus, the ULW-CCAs could be regarded as composites composed of the BCC matrix, the HCP particles, and the intermetallic compounds. The intermetallic compounds and the HCP particles could be considered as the reinforcements. These reinforcements induce an inhomogeneous deformation behavior, and introduce high dislocation density in the BCC matrix [38]. In this case, the generation of geometrically necessary dislocations is required to accommodate the thermal and elastic-modulus mismatches between the matrix and reinforcements. The strengths of the ULW-CCAs can be defined by [39],

(7) $$\sigma_{com}=\sigma_{m}+∆\sigma ,$$

where *σ_com_* is the strength of the CCAs, *σ_m_* is the strength of matrix, and Δ*σ* is the total increment as a result of the presence of reinforcements.

(8) $$∆\sigma =\sqrt{{\left(∆\sigma_{CTE}\right)}^{2}+{\left(∆\sigma_{EM}\right)}^{2}+{\left(∆\sigma_{Lord}\right)}^{2}+{\left(∆\sigma_{Orowan}\right)}^{2}+{\left(∆\sigma_{Hall-Petch}\right)}^{2}},$$

where Δ*σ_CTE_* and Δ*σ_EM_* are the stress increment resulted from the coefficient of thermal expansion, and the elastic-modulus mismatch between the reinforcements and the metallic matrix, respectively. Δ*σ_Lord_* highly depends on the interfacial bonding between the matrix and the reinforcement. Δ*σ_Orowan_* results from the dislocation loops that are formed when the dislocation is bowed and bypasses nanosized reinforcements in the matrix. Δ*σ_Hall-Petch_* is grain-size strengthening. As far as the current ULW-CCAs, the causes for strengthening are multiple.

**Figure 8:**
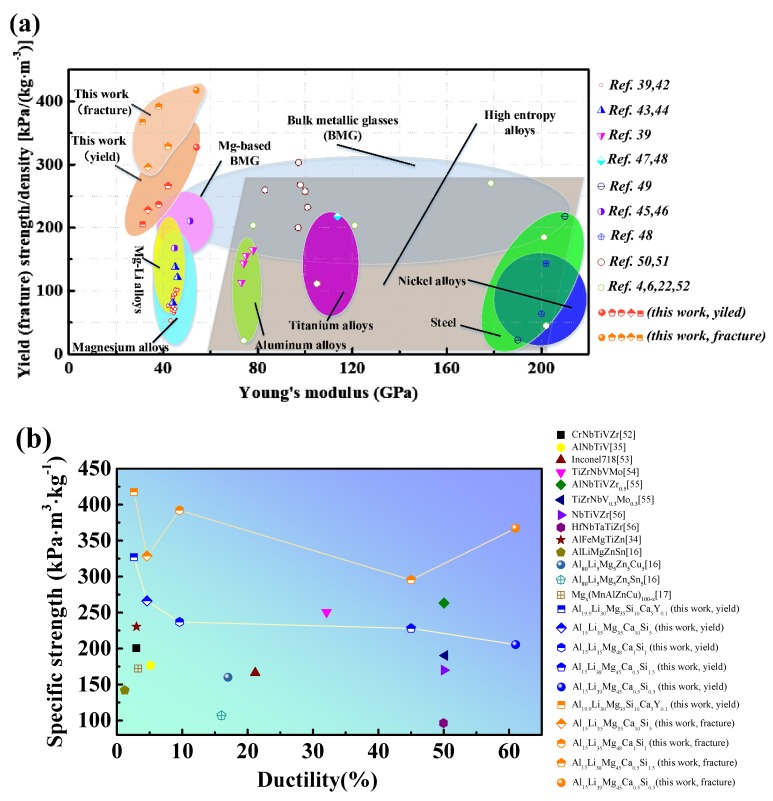
Comparison between five ULW-CCAs and other reported alloys. (**a**) Specific strength as a function of Young’s modulus of five ULW-CCAs and conventional alloys. (**b**) Specific strength vs. ductility of different CCAs.

The Halpin–Tsai equation [40] is used to evaluate the elastic modulus of composites, *E_comp_*, i.e.,

(9) $$E_{comp}=\frac{E_{m}\left(1+2sqV_{r}\right)}{1-qV_{r}},$$

where *q* can be represented by q=ErEm−1ErEm+2s, *E_m_* and *E_r_* represent the elastic moduli of the matrix, and reinforcement, respectively, *V_m_* and *V_r_* are the volume fractions of matrix and reinforcement, respectively, *s* is the aspect ratio of reinforcement. According to the Halpin–Tsai equation, it can be seen that when the volume fraction of the matrix, *V_m_*, is very high, i.e., *V_r_* (= 1 − *V_m_*), the *E_comp_* is close to *E_m_*. It can be seen that the Al_15_Li_35_Mg_48_Ca_1_Si_1_ ULW-CCA shows a low elastic modulus that is as low as the modulus of the matrix. Thus, the elastic moduli of five ULW-CCAs can be calculated by Equation (9) combined with the nanoindentation tests results of each phase. Finally, the elastic moduli of the ULW-CCAs are shown in Figure 8a.

The specific strength vs. Young’s modulus of different alloys is represented in Figure 8a. The CCAs explored in this study are characterized by high specific strengths and low Young’s moduli, as compared with those of other alloys [4,6,22,38,41,42,43,44,45,46,47,48,49,50,51]. The specific strength vs. ductility of the investigated ULW-CCAs is plotted in Figure 8b, which is used to be compared with the other reported CCAs [16,17,34,35,52,53,54,55,56]. It is worth noting that the Al_19.9_Li_30_Mg_35_Si_10_Ca_5_Y_0.1_ ULW-CCA shows the highest specific strength at the expense of ductility. It is well known that LW-CCAs are normally brittle, such as Al_20_Li_20_Mg_10_Sc_20_Ti_30_ [20], Al_20_Be_20_Fe_10_Si_15_Ti_35_ [21], AlLiMgZnSn [16], Mg_x_(MnAlZnCu)_100−x_ [17], and BeCoMgTiZn [19]. However, in the present study, the Al_15_Li_39_Mg_45_Ca_0.5_Si_0.5_ and Al_15_Li_38_Mg_45_Ca_0.5_Si_1.5_ ULW-CCAs display good ductility (Figure 3a and Figure 8b).

## 5. Conclusions

In summary, this work attempts to explore ULW-CCAs with a low elastic modulus, high strength, and good ductility. Some low density and low Young’s modulus elements are used to design the ULW-CCAs according to multi-dimensional phase diagrams, and the strategy of designing HEAs. An outstanding fracture strength of ~710 MPa and an excellent yield strength of ~556 MPa are achieved in the Al_19.9_Li_30_Mg_35_Si_10_Ca_5_Y_0.1_ ULW-CCA although it shows a tiny plastic strain of ~2.6%. By adjusting the composition of the CCAs, the Al_15_Li_35_Mg_48_Ca_1_Si_1_ ULW-CCA with the good compressive plasticity is successfully developed. The microstructure of the Al_15_Li_35_Mg_48_Ca_1_Si_1_ ULW-CCA suggests that a high hardness in the intermetallic compounds and the submicron-sized particles are favorable for enhancing the strength. The volume fraction of the BCC solid solution plays a dominative role in increasing the compressive strain. The Al_15_Li_38_Mg_45_Ca_0.5_Si_1.5_ and Al_15_Li_39_Mg_45_Ca_0.5_Si_0.5_ ULW-CCAs exhibit good plasticity of >45%, and >60%, respectively. These ULW-CCAs show the high specific strength, good ductility, and low Young’s modulus, as compared with the previously reported CCAs.

## Figures and Tables

**Table 1:** The physical and chemical parameters of selected elements.

Parameters	Li	Ca	Mg	Si	Al	Y
Relative atomic mass	6.941	40.08	24.3	28.09	26.98	88.91
Density (g·cm^−3^)	0.54	1.55	1.74	2.33	2.70	4.47
Young’s Modulus (GPa)	4.9	20	45	47	70	64
Melting Point (K)	454	1115	923	1687	933	1799
Crystal Structure	bcc	fcc	hcp	diamond	fcc	hcp
Atomic Radius (pm)	151.9	197.6	160.1	115.3	143.2	180.2
Electronegativity (χ)	0.98	1.00	1.31	1.90	1.61	1.22
Valence Electron Concentration (VEC)	1	2	2	4	3	3

**Table 2:** Theoretical density of the fabricated alloys and the corresponding calculated values for enthalpy of mixing (∆*H_mix_*), ∆*S_mix_*, atomic size difference (*δ*·10), Pauling electronegativity difference (∆*χ*), ratio of entropy to enthalpy values (Ω) and valence electron concentration (*VEC*).

Alloys	*ρ_theory_*	*ρ_experimental_*	∆*H_mix_*	∆*S_mix_*	*δ*·10	∆*χ*	Ω	*VEC*
Al_19.9_Li_30_Mg_35_Si_10_Ca_5_Y_0.1_	1.57	1.70 ± 0.05	−12.6	1.514R	12.05	0.301	0.886	2.12
Al_15_Li_35_Mg_35_Ca_10_Si_5_	1.44	1.57 ± 0.05	−8.95	1.464R	9.590	0.297	1.062	1.90
Al_15_Li_35_Mg_48_Ca_1_Si_1_	1.43	1.52 ± 0.05	−2.71	1.110R	7.108	0.256	4.257	1.82
Al_15_Li_38_Mg_45_Ca_0.5_Si_1.5_	1.40	1.50 ± 0.05	−3.18	1.170R	6.970	0.265	3.875	1.80
Al_15_Li_39_Mg_45_Ca_0.5_Si_0.5_	1.38	1.46 ± 0.05	−2.13	1.075R	4.719	0.263	3.101	1.77

**Table 3:** Chemical compositions (at.%) of phases identified in the microstructures of Al_19.9_Li_30_Mg_35_Si_10_Ca_5_Y_0.1_, Al_15_Li_35_Mg_35_Ca_10_Si_5_, Al_15_Li_35_Mg_48_Ca_1_Si_1_, Al_15_Li_38_Mg_45_Ca_0.5_Si_1.5_, Al_15_Li_39_Mg_45_Ca_0.5_Si_0.5_ ULW-CCAs.

Alloys	Marked Regions	Phase	Al	Mg	Si	Ca
Al_19.9_Li_30_Mg_35_Si_10_Ca_5_Y_0.1_	A	uncertain	86.6	13.4	-	-
	B	CaMgSi	-	30.5	39.4	30.1
	C	uncertain	51.7	28.9	11.9	7.5
	D	Mg_2_Si		61.1	38.9	-
Al_15_Li_35_Mg_35_Ca_10_Si_5_	A	uncertain	22.9	77.1	-	-
	B	Al_2_Ca	57.6	11.2	-	31.1
	C	uncertain	45.4	41.3	13.3	-
	D	CaMgSi	-	38.1	28.3	33.6
Al_15_Li_35_Mg_48_Ca_1_Si_1_	A (Matrix)	uncertain	18	82	-	-
	B (lath-like light)	AlLi	91.2	8.8	-	-
	C (submicron-size particles)	uncertain	12.4	87.6	-	-
	D (lath-like dark)	Li_2_MgAl	55.3	45.7		
Al_15_Li_38_Mg_45_Ca_0.5_Si_1.5_	A	uncertain	12.2	87.8	-	-
	B	AlLi	94.3	5.7	-	-
	C	uncertain	11.6	88.4	-	-
Al_15_Li_39_Mg_45_Ca_0.5_Si_0.5_	A	uncertain	15.7	84.3		
	B	AlLi	93.2	6.8		

## References

[1] Polmear I., StJohn D., Nie J.-F., Qian M. (2017). Light Alloys: Metallurgy of the Light Metals.

[2] Li H.F., Xie X.H., Zhao K., Wang Y.B., Zheng Y.F., Wang W.H., Qin L. (2013). In vitro and in vivo studies on biodegradable CaMgZnSrYb high-entropy bulk metallic glass. Acta Biomater..

[3] Yeh J.W., Chen S.K., Lin S.J., Gan J.Y., Chin T.S., Shun T.T., Tsau C.H., Chang S.Y. (2004). Nanostructured high-entropy alloys with multiple principal elements: Novel alloy design concepts and outcomes. Adv. Eng. Mater..

[4] Miracle D.B., Senkov O.N. (2017). A critical review of high entropy alloys and related concepts. Acta Mater..

[5] Gorsse S., Miracle D.B., Senkov O.N. (2017). Mapping the world of complex concentrated alloys. Acta Mater..

[6] Gao M.C., Yeh J.-W., Liaw P.K., Zhang Y. (2016). High-Entropy Alloys.

[7] Gorsse S., Couillaud S., Gaudin E., Bobet J.L. (2017). Physical properties of the multifunctional Mg_80_Ni_10_Gd_10_ alloy. Mater. Sci. Eng. A.

[8] Gludovatz B., Hohenwarter A., Catoor D., Chang E.H., George E.P., Ritchie R.O. (2014). A fracture-resistant high-entropy alloy for cryogenic applications. Science.

[9] Lu Z.P., Wang H., Chen M.W., Baker I., Yeh J.W., Liu C.T., Nieh T.G. (2015). An assessment on the future development of high-entropy alloys: Summary from a recent workshop. Intermetallics.

[10] Ye Y.F., Wang Q., Lu J., Liu C.T., Yang Y. (2016). High-entropy alloy: Challenges and prospects. Mater. Today.

[11] Zhou Y., Zhang Y., Wang Y., Chen G. (2007). Solid solution alloys of AlCoCrFeNiTi_x_ with excellent room-temperature mechanical properties. Appl. Phys. Lett..

[12] Wu S.W., Wang G., Yi J., Jia Y.D., Hussain I., Zhai Q.J., Liaw P.K. (2017). Strong grain-size effect on deformation twinning of an Al_0.1_CoCrFeNi high-entropy alloy. Mater. Res. Lett..

[13] Hemphill M.A., Yuan T., Wang G.Y., Yeh J.W., Tsai C.W., Chuang A., Liaw P.K. (2012). Fatigue behavior of Al_0.5_CoCrCuFeNi high entropy alloys. Acta Mater..

[14] Tang Z., Yuan T., Tsai C.-W., Yeh J.-W., Lundin C.D., Liaw P.K. (2015). Fatigue behavior of a wrought Al_0.5_CoCrCuFeNi two-phase high-entropy alloy. Acta Mater..

[15] Lee C., Chen Y., Hsu C., Yeh J., Shih H. (2007). The effect of boron on the corrosion resistance of the high entropy alloys Al_0.5_CoCrCuFeNiB_x_. J. Electrochem. Soc..

[16] Yang X., Chen S., Cotton J., Zhang Y. (2014). Phase stability of low-density, multiprincipal component alloys containing aluminum, magnesium, and lithium. JOM.

[17] Li R., Gao J.C., Fan K. (2010). Study to microstructure and mechanical properties of Mg containing high entropy alloys. Mater. Sci. Forum.

[18] Li R., Gao J.C., Fan K. (2011). Microstructure and mechanical properties of MgMnAlZnCu high entropy alloy cooling in three conditions. Mater. Sci. Forum.

[19] Chen Y.-L., Tsai C.-W., Juan C.-C., Chuang M.-H., Yeh J.-W., Chin T.-S., Chen S.-K. (2010). Amorphization of equimolar alloys with HCP elements during mechanical alloying. J. Alloys Compd..

[20] Youssef K.M., Zaddach A.J., Niu C.N., Irving D.L., Koch C.C. (2015). A Novel Low-Density, High-Hardness, High-entropy Alloy with Close-packed Single-phase Nanocrystalline Structures. Mater. Res. Lett..

[21] Tseng K., Yang Y., Juan C., Chin T., Tsai C., Yeh J. (2018). A light-weight high-entropy alloy Al_20_Be_20_Fe_10_Si_15_Ti_35_. Sci. China-Technol. Sci..

[22] Zhang Y., Zuo T.T., Tang Z., Gao M.C., Dahmen K.A., Liaw P.K., Lu Z.P. (2014). Microstructures and properties of high-entropy alloys. Prog. Mater. Sci..

[23] Senkov O., Miller J., Miracle D., Woodward C. (2015). Accelerated exploration of multi-principal element alloys for structural applications. Calphad.

[24] Sun W., Huang X., Luo A.A. (2017). Phase formations in low density high entropy alloys. Calphad.

[25] Feng R., Gao M.C., Lee C., Mathes M., Zuo T., Chen S., Hawk J.A., Zhang Y., Liaw P.K. (2016). Design of light-weight high-entropy alloys. Entropy.

[26] Miracle D.B., Miller J.D., Senkov O.N., Woodward C., Uchic M.D., Tiley J. (2014). Exploration and development of high entropy alloys for structural applications. Entropy.

[27] Yang X., Zhang Y. (2012). Prediction of high-entropy stabilized solid-solution in multi-component alloys. Mater. Chem. Phys..

[28] Zhang Y., Lu Z., Ma S., Liaw P., Tang Z., Cheng Y., Gao M. (2014). Guidelines in predicting phase formation of high-entropy alloys. MRS Commun..

[29] Yeh J.-W. (2013). Alloy design strategies and future trends in high-entropy alloys. JOM.

[30] Zhang Y., Yang X., Liaw P. (2012). Alloy design and properties optimization of high-entropy alloys. JOM.

[31] Guo S., Hu Q., Ng C., Liu C. (2013). More than entropy in high-entropy alloys: Forming solid solutions or amorphous phase. Intermetallics.

[32] Sheng G., Liu C.T. (2011). Phase stability in high entropy alloys: Formation of solid-solution phase or amorphous phase. Prog. Nat. Sci..

[33] Zhang Y., Zhou Y.J., Lin J.P., Chen G.L., Liaw P.K. (2008). Solid-solution phase formation rules for multi-component alloys. Adv. Eng. Mater..

[34] Hammond V.H., Atwater M.A., Darling K.A., Nguyen H.Q., Kecskes L.J. (2014). Equal-channel angular extrusion of a low-density high-entropy alloy produced by high-energy cryogenic mechanical alloying. JOM.

[35] Stepanov N., Shaysultanov D., Salishchev G., Tikhonovsky M. (2015). Structure and mechanical properties of a light-weight AlNbTiV high entropy alloy. Mater. Lett..

[36] Stepanov N., Yurchenko N.Y., Shaysultanov D., Salishchev G., Tikhonovsky M. (2015). Effect of Al on structure and mechanical properties of AlxNbTiVZr (x = 0, 0.5, 1, 1.5) high entropy alloys. Mater. Sci. Technol..

[37] Fischer-Cripps A.C. (2011). Nanoindentation testing. Nanoindentation.

[38] Gupta M., Ling S.N.M. (2011). Magnesium, Magnesium Alloys, and Magnesium Composites.

[39] Goh C., Wei J., Lee L., Gupta M. (2007). Properties and deformation behaviour of Mg-Y_2_O_3_ nanocomposites. Acta Mater..

[40] Wong W.L.E., Karthik S., Gupta M. (2013). Development of high performance Mg-Al_2_O_3_ composites containing Al_2_O_3_ in submicron length scale using microwave assisted rapid sintering. Mater. Sci. Technol..

[41] Brook G. (1998). Smithells Light Metals Handbook.

[42] Totten G.E., Xie L., Funatani K. (2003). Handbook of Mechanical Alloy Design.

[43] Kainer K.U., Mordike B.L. (2000). Magnesium Alloys and Their Applications.

[44] Xi X., Wang R., Zhao D., Pan M., Wang W. (2004). Glass-forming Mg-Cu-RE (RE = Gd, Pr, Nd, Tb, Y, and Dy) alloys with strong oxygen resistance in manufacturability. J. Non-Cryst. Solids.

[45] Yuan C., Xi X. (2011). On the correlation of Young’s modulus and the fracture strength of metallic glasses. J. Appl. Phys..

[46] Welsch G., Boyer R., Collings E. (1993). Materials Properties Handbook: Titanium Alloys.

[47] Handbook M. (1990). Properties and Selection: Nonferrous Alloys and Special-Purpose Materials.

[48] Aggen G., Allen M. (2018). ASM Handbook Volume I Properties and Selection: Irons, Steels, and High-Performance Alloys.

[49] Trexler M.M., Thadhani N.N. (2010). Mechanical properties of bulk metallic glasses. Prog. Mater. Sci..

[50] Szuecs F., Kim C., Johnson W. (2001). Mechanical properties of Zr_56.2_Ti_13.8_Nb_5.0_Cu_6.9_Ni_5.6_Be_12.5_ ductile phase reinforced bulk metallic glass composite. Acta Mater..

[51] Senkov O., Wilks G., Miracle D., Chuang C., Liaw P. (2010). Refractory high-entropy alloys. Intermetallics.

[52] Senkov O., Senkova S., Miracle D., Woodward C. (2013). Mechanical properties of low-density, refractory multi-principal element alloys of the Cr-Nb-Ti-V-Zr system. Mater. Sci. Eng. A.

[53] Dudzinski D., Devillez A., Moufki A., Larrouquere D., Zerrouki V., Vigneau J. (2004). A review of developments towards dry and high speed machining of Inconel 718 alloy. Int. J. Mach. Tools Manuf..

[54] Wu Y.D., Cai Y.H., Chen X.H., Wang T., Si J.J., Wang L., Wang Y.D., Hui X.D. (2015). Phase composition and solid solution strengthening effect in TiZrNbMoV high-entropy alloys. Mater. Des..

[55] Stepanov N.D., Yurchenko N.Y., Sokolovsky V.S., Tikhonovsky M.A., Salishchev G.A. (2015). An AlNbTiVZr_0.5_ high-entropy alloy combining high specific strength and good ductility. Mater. Lett..

[56] Gao M., Carney C., Doğan Ö., Jablonksi P., Hawk J., Alman D. (2015). Design of refractory high-entropy alloys. JOM.

